# Field Crickets Compensate for Unattractive Static Long-Distance Call Components by Increasing Dynamic Signalling Effort

**DOI:** 10.1371/journal.pone.0167311

**Published:** 2016-12-09

**Authors:** Emily M. McAuley, Susan M. Bertram

**Affiliations:** 1 Department of Biological Sciences, Simon Fraser University, Burnaby, British Columbia, Canada; 2 Department of Biology, Carleton University, Ottawa, Ontario, Canada; University of Melbourne, AUSTRALIA

## Abstract

The evolution of multiple sexual signals presents a dilemma since individuals selecting a mate should pay attention to the most honest signal and ignore the rest; however, multiple signals may evolve if, together, they provide more information to the receiver than either one would alone. Static and dynamic signals, for instance, can act as multiple messages, providing information on different aspects of signaller quality that reflect condition at different time scales. While the nature of static signals makes them difficult or impossible for individuals to augment, dynamic signals are much more susceptible to temporary fluctuations in effort. We investigated whether male Texas field crickets, *Gryllus texensis*, that produce unattractive static signals compensate by dynamically increasing their calling effort. Our findings lend partial support to the compensation hypothesis, as males that called at unattractive carrier frequencies (a static trait) spent more time calling each night (a dynamic trait). Interestingly, this finding was most pronounced in males that called with attractive pulse characteristics (static traits) but did not occur in males that called with unattractive pulse characteristics. Males that signalled with unattractive pulse characteristics (duration and pause) spent less time calling through the night. Our correlative findings on wild caught males suggest that only males that signal with attractive pulse characteristics may be able to afford to pay the costs of both trait exaggeration and increased calling effort to compensate for poor carrier frequencies.

## Introduction

Multiple sexual signals occur across a broad range of taxa, from mammals, birds and fish, to crustaceans, arachnids and insects. These signals can be present as multiple, discrete sexual traits or as complex signals that are made up of several independent components occurring in either the same sensory modality (multicomponent signals) or multiple sensory modalities (multimodal signals [1]; reviewed in [2]). Conspecific receivers use these signals to evaluate a signaller’s potential as a mate (or competitor). Due to the energetic costs involved in trait production, maintenance and expression, sexual signals are often highly condition-dependent; therefore, only high quality individuals should be able to afford the higher costs required to exaggerate their sexual signals. As such, sexual signals should honestly indicate signaller quality and the benefits they are able to offer to a potential mate [3–5].

However, honest signalling theory appears inconsistent with the evolution of multiple sexual traits, largely because receivers should favour the most honest signal and ignore the rest [6–8]. Signals that do not accurately reflect mate quality (unreliable signals) should not persist unless they are relatively inexpensive to produce, have no fitness costs and remain slightly attractive to potential mates [9]. Nonetheless, multiple sexual traits may evolve if, together, they provide more information about mate quality than a single trait alone [10]. This can occur if multiple traits increase receiver fitness by reducing mate choice errors or the costs of choice [10]. The multiple messages hypothesis suggests that multiple traits reflect different aspects of signaller quality, responding to different environmental or genetic factors [7, 9, 10]. Receivers can evaluate multiple messages together, to gain a more accurate estimate of overall mate quality, or can evaluate signals separately, thereby assessing the aspects of quality in which they are most interested [10].

Multiple traits have the potential to reflect different aspects of signaller quality that affect condition at different time scales. Static signals change relatively little over time (have low coefficients of variation) and are generally produced by structures that are “fixed” or are relatively unaffected by short-term fluctuations in the internal or external environment, suggesting static sexual traits may be reflective of condition on a longer time scale (either due to genetic quality or early life condition, etc. [2, 10, 11]. Conversely, dynamic signals change more rapidly over time (have high coefficients of variation) and generally consist of behaviours that respond quickly to fluctuations in intrinsic or extrinsic factors, suggesting dynamic sexual traits may be reflective of short-term fluctuations in current condition [2, 10, 11]. For example, feather brightness in bowerbirds is a static sexual trait, changing only when an individual moults. As such, feather brightness is a suitable honest indicator of endoparasite load, a static measure of condition that also fluctuates at a relatively slow rate over time [12]. Conversely, bower quality is a dynamic signal that can change by the hour as the bird adds, subtracts or rearranges the decorations. Bower quality accurately reflects ectoparasite load, a relatively dynamic measure of condition, which can also change quickly with preening or bathing [12].

**Fig 1:**
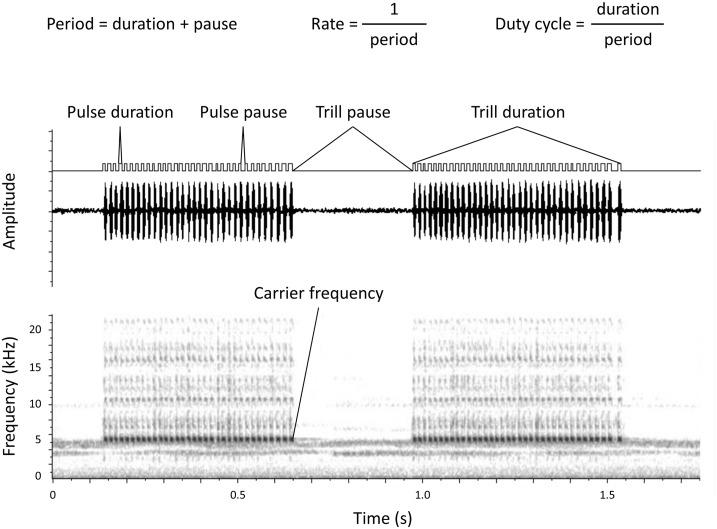
Components of the male acoustic mate attraction signal in the Texas field cricket, *G*. *texensis*.

Here, we explore whether individuals compensate for poor long-term static signals by increasing their investment in more variable short-term dynamic signals using a wild-caught population of Texas field crickets, *Gryllus texensis*. Adult male field crickets produce long-distance acoustic mate attraction signals (calls) by rubbing their modified forewings together. When a male closes his wings, the scraper of one wing hits the teeth of the file on the other wing which causes the harp to resonate, producing a single pulse of sound [13–15]. Males concatenate these pulses into trills (Fig 1; [16, 17]) and trills are concatenated into calling bouts. As such, long distance acoustic mate attraction signals are multicomponent sexual traits that consist of several temporal and spectral components (Fig 1).

In general, signal composition is determined by static traits, while measures of signalling effort are more dynamic [18]. This holds true in Texas field crickets, as carrier frequency, sound pressure level (amplitude), and pulse components (duration, pause, period, rate, and duty cycle) all have low coefficients of variation (CV<18; Table 1; *sensu* [11]), whereas trilling components (duration, pause, period, rate, and duty cycle) and calling effort all have high coefficients of variation CV>25; Table 1; *sensu* [11]). Calling effort is the most dynamic trait of all, as it varies throughout the day, as well as from one day to the next, and has the highest coefficient of variation (average = 140; Table 1). Note, however, that the categorization of traits as static or dynamic is species specific, as pulses per trill and trill duration tend to be very stereotyped (static) within cricket species that chirp, but extremely variable (dynamic) within cricket species that trill (e.g. [19, 20]).

**Table 1:** Coefficients of variation for call characteristics from previous studies on *G*. *texensis*.

Call Parameter	CV%	Classification	N	Ref
Carrier Frequency	4		37	[21]
	5		63	[22]
	4		25	[23]
Average	4	Static		
Amplitude	18		63	[22]
	16		25	[23]
Average	17	Static		
Pulse Duration	14		37	[21]
	15		63	[22]
	17		25	[23]
Average	15	Static		
Pulse Pause	13		37	[21]
	10		25	[23]
Average	12	Static		
Pulse Period	6		37	[21]
	11		25	[23]
Average	9	Static		[24]
Pulse Rate	6		37	[21]
	11		25	[23]
	9		132	[23]
Average	9	Static		
Pulse Duty Cycle	14		37	[21]
	14		25	[23]
Average	14	Static		
Trill Duration	46		37	[21]
	58		63	[22]
	52		25	[23]
	40		144	[24]
Average	49	Dynamic		
Trill Pause	46		37	[21]
	73		63	[22]
	39		25	[23]
	28		142	[24]
Average	47	Dynamic		
Trill Period	38		37	[21]
	31		25	[23]
Average	35	Dynamic		
Trill Rate	28		37	[21]
	25		25	[23]
Average	27	Dynamic		
Trill Duty Cycle	19		37	[21]
	33		25	[23]
Average	26	Dynamic		
Bout Duration	173	Dynamic	20	[25]
Bout Number	208	Dynamic	20	[25]
Time Spent Calling	187		63	[22]
	165		35	[23]
	86		20	[25]
	122		55	[25]
Average	140	Dynamic		

Many of these temporal and spectral components are affected by different aspects of male condition. For example, in the European field cricket, *G*. *campestris*, chirp rate is affected by adult condition [26], while carrier frequency is affected by juvenile condition [27]. In the Jamaican field cricket, *G*. *assimilis*, juvenile condition is signalled by pulse pause, pulse rate and chirp duration, while adult condition is signalled by carrier frequency [28]. In the western stutter-trilling cricket, *G*. *integer*, adult males that were fasted for two days experienced decreases in body condition and the duration of their trills [29]. Moreover, the decrease in body mass was proportional to the decrease in trill duration, suggesting that trill duration honestly reflects condition [29].

Female crickets exhibit distinct preferences for many components of male acoustic mate attraction signals, most of which involve an increase in the male’s energetic investment [30]. For example, female *G*. *texensis* generally prefer calls with a higher trill duty cycle [21], female *G*. *lineaticeps* prefer calls that have high chirp rates and long chirp durations [30], female *G*. *integer* prefer long call trill durations [31, 32], and female *G*. *campestris* prefer calls of low carrier frequency [33, 34]. Females also passively select for males that spend more overall time calling [35–39]. For example, male *G*. *texensis* that spend the most time calling throughout the night are more likely to attract females to mate with them [35].

We obtained quantitative measures of several static call components (carrier frequency, amplitude, and pulse characteristics, all related to call composition) as well as three measures of dynamic call components related to overall calling effort. We hypothesized that males would compensate for poor static signals. We assumed that compensation in this system requires that individuals cannot change their initial development/investment in the static signal, and that they later evaluate their own static signal quality (perhaps through visitation rate) and adjust their investment into dynamic signals accordingly. We therefore predicted that some of the static signals would correlate with adult body size, and therefore be indicative of condition during development. We also predicted that males with poor static signals would increase their investment in dynamic signals by increasing their calling effort. To our knowledge, ours is the first animal study to investigate this possibility.

## Materials and Methods

Our study was conducted in accordance with the guidelines of the Canadian Council on Animal Care and we did not require specific permits to collect crickets or conduct our experiments. Adult male *G*. *texensis* of unknown age, calling history, and mating experience that were attracted to bright lights were collected at night from the surrounding lighted areas (fields and parking lots) in Smithville, Bastrop County, Texas, United States (latitude ~ 30° 17’ N, longitude ~ 97° 46’ W, elevation ~145 m) from Sept 26-Oct 4, 2007. Males were brought back to the Stengl Lost Pines Biological Station and placed into individual 500 ml plastic containers, each with a 30 ml plastic cup filled with gravel and water, and a section of cardboard egg carton for shelter. They were fed powdered rat chow (Harland Teklad Rodent Diet no. 8604; 24.3% protein, 40.2% carbohydrate, 4.7% lipid, 16.4% fiber, 7.4% ash) and given water *ad libitum*.

Individual recordings to assess spectral and temporal call composition were obtained between 20:00 h and 06:00 h at a temperature ranging from 23.9 to 26.5°C (*X* ∓ *SD* = 25.1 ∓ 0.6°C). Males that were observed to produce acoustic mate attraction calls (N = 30) were recorded for at least two minutes using a handheld Zoom Handy Recorder H4 (Zoom Corporation, Japan). The microphone of the recorder was placed approximately 3 cm from the exterior of the plastic container (i.e. within approximately 7 ± 4 cm from the cricket, depending on the cricket’s location in the container). Recording occurred from outside the containers because the containers were too small in which to fit the Zoom recorders. We assumed that any distortion that occurred as a result of recording outside the plastic container would have occurred across all recordings, as all males were housed in identical plastic containers.

As soon as males had their individual call components successfully recorded, they were relocated outside of the recording room. We removed recorded males from the room to maximize our opportunities to record males that rarely called, as we wanted to minimize our bias against low-effort signallers. We completed all recordings within four nights of capture (range = 0 to 4 days; (*X* ∓ *SD* = 1.6 ∓ 1.0 *days*), and assumed that any male that did not call within four nights was a non-calling male. Over 90% of males called within the first two nights of capture. However, because our recording period was up to four nights long, the few males that took longer to call had more access to high quality food than males that called early on. While this trade-off has the potential to partially obscure the relationship with dynamic song characteristics, we felt it was more important to not bias our dataset against low-effort signalers.

Males who had their individual call components successfully recorded the previous evening were then placed in the Electronic Acoustic Recorders system (EARs; [40]) to have their calling effort assessed. We completed this two-step process (hand-held recording the spectral properties and then using the EARs to record calling effort) because our EARs unit samples at such a slow rate (eight times per second) that it can only quantify whether a male is calling or not each second; it is incapable of quantifying any of the spectral properties of the call. With the EARs second by second calling data we were able to ascertain the times that each male started calling, stopped calling, the duration of his calling bouts and breaks, and his overall time spent calling. When in the EARs unit, each individual’s container 30 ml plastic container was surrounded by 2.5 cm thick acoustic foam to reduce sound contamination from the surrounding environment and neighbouring males. A microphone was hung inside each male’s container, approximately 5 cm above the cricket. Males were left in the EARs for at least 24 h (*X* ∓ *SD* = 62.4 ∓ 29.3*h*).

Upon removal from the EARs, males were euthanized and then stored at -15°C. The frozen bodies were packed in ice and flown to Carleton University, Ottawa, ON, Canada (Canadian Food Inspection Agency import permit number 2007–03130). We then obtained post-mortem measures of head width, pronotum width, pronotum length, pronotum area and dry mass for each recorded male (N = 30). Body size measurements were obtained from highly magnified photographs taken with a Zeiss Axio Observer inverted microscope of the frozen bodies using AxioVision Software (Carl Zeiss, Jena, Germany).

### Quantification of Call Composition and Effort

We used Spike2 audio software (Cambridge Electronic Design Ltd., Cambridge, U.K.) to analyze the components of each individual handheld recording. For each male, a total recording time of 1–2 min was analyzed, either from a single continuous recording, or from two or three separate recordings. Males were recorded until a total time of two minutes was reached, at which point the recording was shut off. Recordings were not filtered in any way prior to analysis. Spike2 audio software was used to create an “on/off” trace of the call (Fig 1) in order to determine when a male was producing a pulse of sound or not based on an amplitude threshold. In all cases, the “trace” of the call produced by Spike2 was compared visually to the actual waveform of the call to ensure that each pulse of sound was being accurately captured. This threshold was the same in every case, except for four individuals which were relatively quiet callers; we lowered the threshold to a detectable value for these four males. Spike2 was then used to calculate the mean values for five static measures of call composition for which female *G*. *texensis* are known to select [21]: pulse duration, pulse pause, pulse rate, pulse duty cycle and carrier frequency (Fig 1; Table 1).

We used a Matlab (The Mathworks Inc., Natick, Massachusetts, U.S.A.) script to summarize the raw EARs data into three dynamic measures of calling effort: calling bout duration, bout number and time spent calling. We quantified calling bout duration as the average length of time that the male called continuously without taking at least a 1 min break, bout number as the average number of bouts per night and time spent calling as the total number of minutes spent signalling each night [40] As such, time spent calling is equal to the product of bout duration and bout number. We categorized these traits as dynamic *a priori* because time spent calling exhibits the highest coefficient of variation (average = 145; Table 1) of any calling trait measured in *G*. *texensis*.

*Gryllus texensis* and *G*. *rubens* are morphologically cryptic and best separated by song (most notably pulse rate, trill duration, trill rate, and carrier frequency). Given this, we asked whether any of our *G*. *texensis* males could have been misidentified. Two males had low pulse rates for *G*. *texensis* (<56 pulses per second) suggesting they might be misidentified (*sensu* [18], Fig 1a). However, both males called at carrier frequencies above 5.5 kHz, with trill durations about 400 ms, and trill rates above 1.2 trills/second, suggesting they are *G*. *texensis* (*sensu* [18], Fig 1c and 1d, respectively). Further, given our study site was located about 100km west of the most western end of the range of *G*. *rubens*, we opted to include these males in our analyses.

### Statistical Analyses

All statistical analyses were performed with JMP 11 statistical software (SAS Institute Inc., Cary, North Carolina, U.S.A.). Because size variables were highly intercorrelated, we performed a principal component analysis on all four size variables (head width, pronotum width, pronotum length, pronotum area) to obtain a single measure of body size (PC Size). Our principal component analysis revealed that all body size measures were highly positively correlated. Each measure loaded relatively equally onto the one orthogonal principal component (eigenvector loadings ranged from 0.472–0.523); PC Size described 87% of the overall variation in body size (eigenvalue = 3.47).

We ran linear regressions of each static call component for which females are known to select [21] onto body size (PC Size). We used Goodness of Fit to test for normality of residuals and then Box Cox transformed pulse pause and pulse rate to meet normality assumptions. All models fit assumptions of homoscedasticity by visual inspection of Q-Q plots. We then quantified the attractiveness of each male’s call component using information from a female preference study on *G*. *texensis* [21]. We used the plots of female preference score versus call components from [21] to create female preference functions for each of our static signals. We then used the resulting female preference functions to compute the attractiveness for each of our observed individual male’s static call components. Note: we feel confident using the female preferences plots to quantify male attractiveness in our study because most crickets from Blankers et al. [21] study were collected within 100km of our crickets and our two studies were conducted at the same temperature within the same temperature range (average = 25°C, range = 24–26°C).

**Fig 2:**
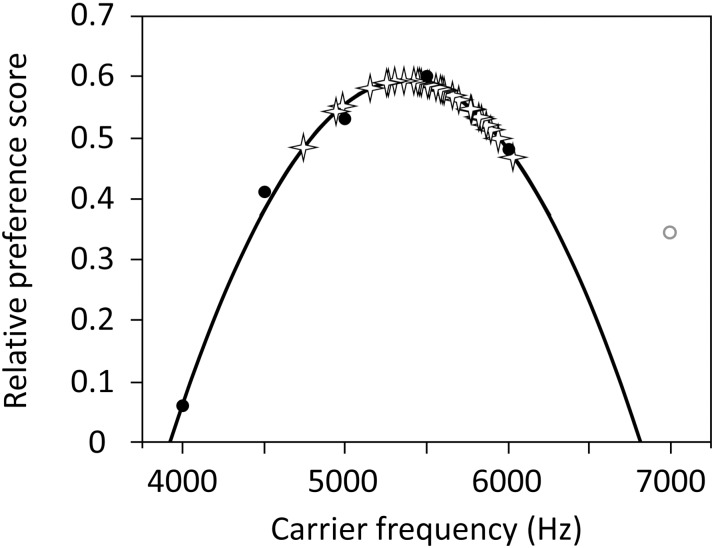
Female preference for male calls based on carrier frequency. Black circles represent the means from a female preference study using *G*. *texensis* [21]. We fit a quadratic equation (solid line) to the preference scores for the carrier frequencies from 4000–6000 Hz (black circles). The preference score for 7000 Hz (open grey circle) was not included because no males in this study or in Blankers et al. [21] were observed to call at a carrier frequency that high and including it made the quadratic function fit less tightly with the other data points. Stars indicate the carrier frequencies of males observed in this study (n = 30).

We used the mean female preference scores for carrier frequencies from 4000 to 6000 Hz from Fig 2a in Blankers et al. [21] to create a quadratic equation representing female preference for carrier frequency (Fig 2). We excluded the female preference score for 7000 Hz because none of the males that we nor Blankers et al. [21] observed exhibited frequencies that high (our max = 6031 Hz) and including the female preference score for calls at 7000 Hz made the curve fit the other values less tightly.

**Fig 3:**
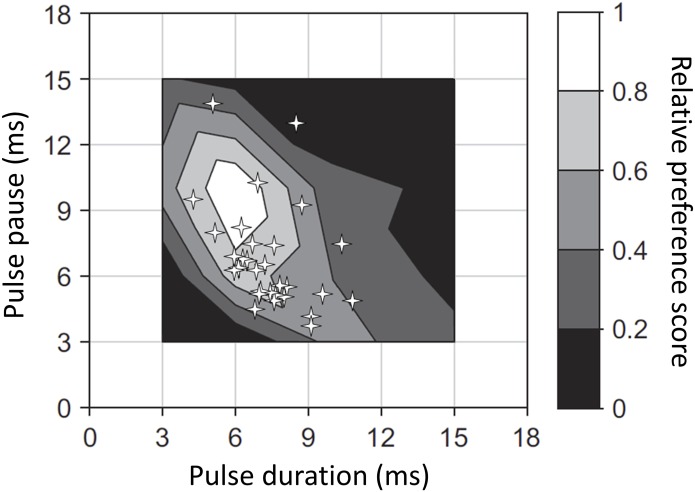
Observed male pulse durations and pauses from this study (stars; n = 30) overlaid onto a bivariate plot of female *G*. *texensis* preference scores from Fig 3b in Blankers et al. [21]. Males were assigned the highest female preference score for the contour plot in which they were found. For example, the data point in the darkest contour (0–0.2) was assigned a preference score of 0.2. Points in the next darkest contour (0.2–0.4) were assigned scores of 0.4, and so on.

For the pulse characteristics (duration, pause and duty cycle), we used Fig 3a from Blankers et al. [21], a bivariate plot of female preference for both pulse duration and pause, to assign female preference scores. We calculated female preference scores (Fig 3) based on a combination of both pulse duration and pulse pause because we identified both pulse duration and pulse pause as static traits (significant association with body size; p = 0.0010 and p = 0.0270, respectively).

We then investigated whether males that exhibited unattractive static signals exhibited higher calling effort than males that exhibited attractive static signals. To do this, we created standard least squares regressions to determine whether three measures of calling effort (mean nightly time spent calling, number of calling bouts and calling bout duration, all of which are assumed to be dynamic signals) were affected by the attractiveness of the static signals (carrier frequency and pulse characteristics) and their interaction. We used Goodness of Fit to test the residuals for normality and then Box Cox transformed calling bout duration to meet assumptions of normality. All models fit the assumptions of homoscedasticity by visual inspection of Q-Q plots and residuals were fairly evenly distributed around a mean of zero.

## Results

We examined the relationship between each static call component for which females are known to select [21] and adult body size (Table 2). Since adult body size is determined during development, any calling component that is consistently correlated with body size across males should also be affected by long-term juvenile condition. Carrier frequency, pulse duration, pulse pause and pulse duty cycle were all significantly correlated with body size (Table 2).

**Table 2:** Linear relationships between pulse characteristics for which females are known to select [21] and male body size (n = 30).

Call component	Intercept ± SE	Estimate ± SE	R^2^_adj_	F	p
Carrier frequency (Hz)	5527±53	-66.9±28.9	0.131	5.38	0.0279*
Pulse duration (ms)	7.29±0.23	0.460±0.125	0.301	13.5	0.0010*
Pulse pause Box Cox (ms)	27.7±0.33	-0.422±0.181	0.133	5.45	0.0270*
Pulse rate Box Cox (min^-1^)	37.2±1.7	-0.161±0.951	-0.035	0.029	0.8667
Pulse duty cycle	0.524±0.017	0.0306±0.0092	0.256	11.0	0.0026*

(*) Denotes p < 0.03, the Benjamini and Hochberg False Discovery Rate corrected significance level used to reduce artificial inflation of Type 1 error rates.

**Fig 4:**
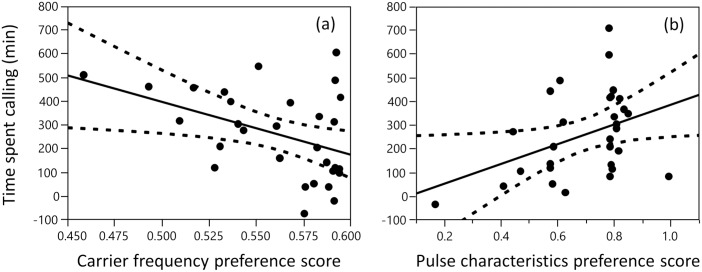
Leverage plots of the effects of the female preference scores for the static call components. (a) carrier frequency, and (b) pulse characteristics (duration and pause) on the amount of time males spent calling each night. Dashed lines represent 95% confidence intervals.

We then assigned female preference scores for each male’s static call components based on a female *G*. *texensis* preference study [21]. There was a significant negative relationship between the attractiveness of carrier frequency and time spent calling; males that called at unattractive carrier frequencies spent more time calling than males that called at attractive carrier frequencies (Fig 4a; Table 3). However, the opposite was observed for unattractive pulse characteristics; males spent significantly more time calling per night the more attractive their pulse duration and pause were (Fig 4b; Table 3).

**Table 3:** Standard least squares regressions of time spent calling and calling bout number onto static signal component attractiveness.

Calling Effort	Term	R^2^_adj_	Estimate	SE	F/t	P
Time spent calling (min)	Model	0.217			3.69	0.0246*
	Intercept		1235	520	2.38	0.0251*
	Carrier frequency score		-2237	924	-2.42	0.0228*
	Pulse characteristics score		417.7	191.1	2.19	0.0381
	Frequency score x pulse score		-7722	5150	-1.50	0.1458
Number of calling bouts per night	Model	0.310			5.35	0.0053*
	Intercept		42.66	12.77	3.34	0.0025*
	Carrier frequency score		-66.89	22.73	-2.94	0.0067*
	Pulse characteristics score		8.585	4.700	1.83	0.0792
	Frequency score x pulse score		-328.3	126.6	-2.59	0.0154*
Calling bout duration (min)	Model	-0.051			0.535	0.6622
	Intercept		77.61	51.28	1.51	0.1422
	Carrier frequency score		-100.7	91.22	-1.10	0.2798
	Pulse characteristic score		14.39	18.87	0.76	0.4525
	Frequency score x pulse score		-47.13	508.3	-0.09	0.9268

(*) Denotes the models with p < 0.033, the Benjamini and Hochberg False Discovery Rate corrected significance level used to reduce artificial inflation of Type 1 error rates. DF = 3,26 for each model.

The number of calling bouts a male produced per night was significantly affected by an interaction between carrier frequency and pulse characteristic (duration and pause) attractiveness; when pulse duration and pause were relatively attractive, males that called at unattractive carrier frequencies significantly increased the number of calling bouts they produced in a night (Table 3). However, the more unattractive the pulse characteristics (duration and pause) were, the less steep the relationship between carrier frequency attractiveness and bout number became, eventually becoming slightly positive (i.e. males that called at attractive carrier frequencies slightly increased the number of bouts they produced per night). Neither of the static signals had a significant effect on mean bout duration (Table 3). Thus, males increased their time spent calling by altering the number, but not the duration, of their calling bouts. The latter result was further confirmed by the finding that time spent calling was significantly positively correlated with calling bout number (R^2^_adj_ = 0.369, F = 17.94, p = 0.0002, DF = 29).

## Discussion

Our findings suggest that field-caught male Texas field crickets, *G*. *texensis*, may compensate for a poor long-term static signal by increasing their investment in a dynamic signal. Males that signalled at unattractive carrier frequencies spent more time calling through the night by increasing the number but not the duration of their calling bouts. This finding was most pronounced in males that called with attractive pulse characteristics but did not occur in males that called with unattractive pulse characteristics. Conversely, males that signalled with attractive pulse characteristics spent more time calling through the night. These correlative findings suggest that due to the energetic costs involved in signal production, maintenance and expression, only high quality males that are able to signal with attractive pulse characteristics may be able to afford the higher costs associated with increased calling effort in order to compensate for unattractive carrier frequencies.

Most of the static call components were significantly correlated with body size. In crickets, body size is fixed at adulthood, making it a reliable signal of juvenile condition [21, 28]. We found carrier frequency to be negatively correlated with male body size. This makes sense since the size of a male's harp (the resonant structure found on the male’s forewings) scales with body size. Larger harps vibrate slower than smaller harps, resulting in lower frequency calls [13–15]. Similarly, larger files take more time to scrape across teeth than smaller files, resulting in longer pulse durations [15]; this is consistent with our finding that pulse duration was positively correlated with body size. Conversely, we found pulse pause to be negatively correlated with body size. This may be due to a constraint on pulse rate resulting from female choice rather than from a physical, structural constraint in males. Female *G*. *texensis* prefer a relatively small range of pulse rates relative to other call components [21]; thus, larger males that produce longer pulses must produce proportionately shorter pulse pauses in order to signal at the preferred pulse rate. This is consistent with our findings that pulse duration was positively associated with body size, pulse pause negatively (though not quite significantly), and pulse rate not at all. Together these correlations with body size suggest that these static traits reliably signal juvenile condition.

We found that males calling at unattractive carrier frequencies appeared to compensate by increasing their calling effort, as nightly time spent calling increased with decreasing carrier frequency attractiveness; number of calling bouts produced per night also increased with decreasing carrier frequency attractiveness, particularly if pulse characteristics (duration and pause) were attractive. Carrier frequency constitutes a “first pass” at song recognition; male calls must occur at a carrier frequency that the female tympanic membrane is physically able to hear before the female can assess the other call components, such as pulse characteristics [41]. Female cricket ears are mechanically most receptive to specific frequencies and neural sensitivities drop off quickly at sub-optimal frequencies [42, 43]. While all of the males that we observed called at carrier frequencies near the peak of female *G*. *texensis* responsiveness, some called at carrier frequencies that were less preferred (occurring at frequencies higher or lower than the optimum) than others. Males calling at sub-optimal frequencies are less audible to females, and their calls induce lower neural and phonotactic responses [21, 43]. Additionally, the calls of males made at sub-optimal frequencies have a reduced range, resulting in their being statistically less likely to reach the same number of females than males calling at frequencies of peak female responsiveness, unless males compensate by increasing call amplitude [43]. Enhancing time spent calling when carrier frequency is sub-optimal may be an effective compensation strategy to attract females, especially given male *G*. *texensis* that spend more time calling also call with higher sound pressure levels (correlation = 0.797; [23]).

Several authors have suggested that increasing signalling effort in response to a reduction in future reproductive potential results in dishonest signalling (e.g. [44–46]). However, the males that we observed producing calls at sub-optimal carrier frequencies likely had to spend more time calling to attract a mate than did males calling at the most audible frequencies. According to the terminal investment hypothesis [47–50], males that increase the amount of time they spend calling should concurrently increase their investment in current reproductive opportunities. Further, males that increase their calling effort pay higher costs because calling behaviour is both energetically expensive [22, 51, 52] and takes time away from foraging and other tasks such as intrasexual competition and territory defense. Additionally, males that call more often experience elevated risks of both predation [53] and parasitism [54]. Female parasitoid flies, *Ormia ochracea*, possess unique hearing organs that enable them to use male *Gryllus* mate attraction calls to locate potential hosts [55]. Female *O*. *ochracea* auditory systems are tuned to the carrier frequencies produced by their hosts [55], and flies respond most strongly to calls occurring at carrier frequencies closest to the calls of local *Gryllus* populations [56]. Males that call most often are likely to be more constrained by the threat of parasitism by female flies than males that call less often.

Other non-mutually exclusive hypotheses could explain our finding that males calling at unattractive carrier frequencies increasing their calling effort. For example, some males could have reserved investment in static signals and thus preserved the ability to invest more heavily in dynamic signaling later on (an alternative strategy rather than compensation per se). Alternatively, different combinations of genetic quality and resources could also result in this correlative pattern without clear compensation. Fully differentiating between these different mechanisms requires experimentation, and this is something our lab is currently investigating. If experimental manipulations reveal compensation, then important evolutionary questions can begin to be addressed, such as (1) how dynamic compensation of signaling effort affects sexual selection on multicomponent signals, (2) how such compensation affects selection on female preferences, and (3) does dynamic compensation decouple quality signal-benefit-preference coevolution?

**Fig 5:**
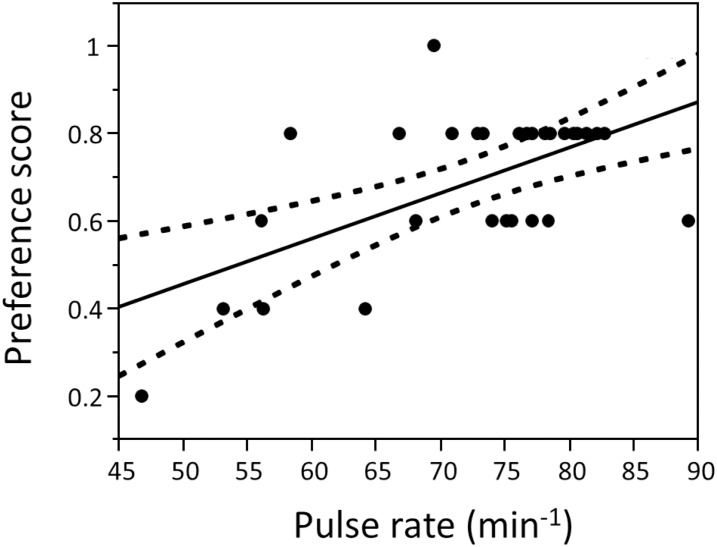
Attractiveness of male pulse rate, in terms of bivariate female preference for pulse characteristics from [21]. Male calls that are more attractive to females have higher pulse rates (R^2^_adj_ = 0.339, F = 15.9, p = 0.0004, DF = 29).

Contrary to our hypothesis that crickets compensate for poor long-term static signals by increasing their investment in more variable short-term dynamic signals, wild-caught males with unattractive pulse characteristics spent less time calling. This finding suggests that pulse characteristics may be indicative of good condition. Males that signal with attractive pulse characteristics may be better able to pay the higher costs of trait exaggeration compared to males that signal with unattractive pulse characteristics. While neither pulse duration nor pulse pause became more exaggerated with increasing attractiveness, together these two variables determine pulse rate, which does increase with pulse characteristic attractiveness (Fig 5). This may be why only males with attractive pulse characteristics seem to be able to compensate for unattractive carrier frequencies by increasing their calling bout numbers, resulting in higher time spent calling. Pulse characteristics may therefore be a condition-dependent handicap ([57] reviewed in [58]; refined from [59, 60]), where the degree of exaggeration seen in male ornaments is assumed to be proportional to the overall condition of the male, such that males in good condition will have more exaggerated ornamentation and higher viability [4, 60–63]. Higher quality males may be able to both (1) acquire the resources necessary to build the structural apparati and maintain the musculature required to produce attractive signals and (2) acquire and maintain the reserves needed to pay the higher energetics costs of calling higher pulse rates and higher calling effort. This could also explain why females closely assess male pulse characteristics [21]. Blankers et al. [21] asserted that after females hear male mate attraction calls (i.e. males call at frequencies the females can hear), the primary set of call characteristics that they assess are pulse characteristics, where they simultaneously evaluate pulse duration and pulse pause, resulting in a pulse rate filter [21].

While our study appears to be the first animal study to examine whether high quality males compensate for unattractive static song components by enhancing their calling effort (a dynamic component), a handful of other field cricket studies support the idea that only high quality males can afford to compensate. For example, a recent study in *G*. *texensis* revealed that attractive males (measured via mating assay, not bioacoustics) increased their calling effort under immune challenge whereas unattractive males did not [64]. Further, male *G*. *integer* who trill for long uninterrupted durations are both most attractive to potential female mates and also most attractive to predators. These males appear to compensate for their elevated predation risk by behaving more cautiously: they take longer to emerge from a safe shelter and they cease calling for longer periods of time after their calls are interrupted compared to males that trill for shorter uninterrupted durations [65].

Even though we found some intriguing patterns, the correlational nature of our study limits our ability to interpret our findings. For example, we found that wild caught males that produced the most attractive pulse characteristics spent the most time calling for females. However, we isolated our males in individual containers and, as a result, the males were unable to attract females. In the wild, males with attractive pulse characteristics are likely to attract more females than males with unattractive pulse characteristics and, once a female is attracted, the male would switch from producing long distance mate attraction calls to producing courtship calls. After mating, would males with the most attractive pulse characteristics go on to call more, as our study suggests? The unknown mating history and social experiences of our wild caught males could also have contributed to the patterns we observed. Perhaps males with attractive call characteristics mated with more females prior to collection, and as a result, altered their subsequent calling effort, explaining our observed patterns. Future research should experimentally manipulate juvenile and adult diet while controlling social experiences and mating history to address whether Texas field crickets compensate for poor static carrier frequencies by increasing their investment in more variable short-term dynamic signals. This is something our lab is currently investigating.

Our findings of partial support for the hypothesis that field crickets compensate for poor long-term static signals by increasing their investment in more variable short-term dynamic signals uphold the theoretical prediction that relationships between sexual advertisement and life history traits can be dynamic (positive or negative; [66–70]). On the one hand, males appear to compensate for unattractive carrier frequencies by increasing their signalling time, especially if they have attractive pulse characteristics. On the other hand, only males with attractive pulse characteristics seem to be able to pay the higher costs associated with signalling more often, suggesting pulse characteristics may honestly indicate signaller quality [3–5]. This may be why females seem to use variation in carrier frequency only as a first-pass indicator of species [41], and use the information associated with variation in pulse characteristics for mate selection [21].
